# Supplementary material for: Work alienation influences nurses’ readiness for professional development and willingness to learn: A cross-sectional correlation study
Source: PLoS One. 2023 May 9;18(5):e0284963. doi: 10.1371/journal.pone.0284963 (PMC10168560; doi:10.1371/journal.pone.0284963)
Supplement: Attachment — Submitted filename: Response to Reviewers.docx [file pone.0284963.s002.docx]

**Response to Reviewers**

Response to the journal requirements

We appreciate your careful review of our manuscript and thank you for the opportunity to submit our revised manuscript and the point-by-point responses to the journal requirements. We hope that we have satisfactorily addressed all issues raised by the journal requirements.

Comment

1. We note that your author list was updated during the revision process. Please complete our Authorship Change Form by following this link: http://journals.plos.org/plosone/s/file?id=13d0/plos-one-change-to-authorship-form.docx. You may return the form as an attachment by emailing plosone@plos.org or by uploading it as a submission file labeled with the file type ‘Other’. Please note that if your manuscript is accepted, we will not be able to complete the publication process without the completed form.

Response

As suggested by Journal Requirement, we completed our Authorship Change Form and uploaded it as a submission file in the revised manuscript.

Dear editor,

We would like to thank you and the reviewer for their valuable comments and feedbacks. Point-by-point responses to reviewers are listed below.

**Reviewer #1**:

Comment 1:

The manuscript is well written and the topic is very interesting because it reflects the work alienation during COVID-19 amongst nurses, and its influence on nurses' readiness for professional development and willingness to learn.

Response 1:

Thank you for your valuable comments.

Comment 2:

I did not assess the statistical analysis rigorously because of my limited strength in quantitative studies.

Response 2:

Thank you for your valuable comments.

Comment 3:

The authors should add the key figures of the results in the abstract section.

Response 3:

As your suggestion, we added key figures to our abstract section.

The authors should use STOBE checklist to check the completeness of their methods section.

Comment 4:

As your suggestion, we added an appendix of STROBE checklist to our revised manuscript.

The authors used a convenience sample. They should add a discussion on the limitation of using the convenience sample.

Response 4:

In response to this comment, we added the convenience sample method in our limitation section. Please see our revised manuscript.

**We hope now that our revised manuscript is acceptable for publication.**
